# Comments on: Lack of association between fat mass and obesity-associated genetic variant (rs8050136) and type 2 diabetes mellitus

**DOI:** 10.15537/smj.2022.43.4.20220218

**Published:** 2022-04

**Authors:** Abdulghani Alsaeed, Amjad M. Yousuf

**Affiliations:** *Department of Endocrine and Diabetes, Prince Sultan Military Medical City, Riyadh, Kingdom of Saudi Arabia*; *Department of Medical Laboratories Technology, College of Applied Medical Sciences, Taibah University, Al Madinah Al Munawarah, Kingdom of Saudi Arabia*

*To the Editor*

I have read with a great interest the article published in the Saudi medical journal by Yousuf et al^1^ with a title Lack of association between fat mass and obesity-associated genetic variant (rs8050136) and type 2 diabetes mellitus. There are several drawbacks on the article for example, there was no point of matching between the health control and the patient’s group. To prove that certain gene is really associated with T2DM (type 2 diabetes mellitus) in a specific population, the healthy controlled group should be matched with patient group for the age, gender, and body mass index which are the risks factors for development of T2DM. All these parameters were highly statistically significant different in the study between the 2 groups. Furthermore, finding similar prevalence of fat mass and obesity-associated gene in the health control and patient group might simply indicate that the chosen control group was at increased risk to develop T2DM. Additionally, the sample size is too small for highly prevalent disease to explore the association of certain gene.

Type 2 diabetes mellitus is a complex syndrome. The hallmark of T2DM is insulin resistance, and relative insulin deficiency. The exact etiology of this disease in most cases is difficult to be revealed. The understanding of the pathogenesis of T2DM has led to innovations in the field of diabetes pharmacology. A proposal of a new terminology called an ominous octet exploring 8 different mechanisms where the current available anti-diabetes medications could work as a pathogenesis for T2DM.^2^ However, T2DM is a multifactorial disease. It usually occurs because of an interaction between a genetic and environmental factor. So, people at risk or genetically susceptible individuals are exposed to acquired environmental factors like obesity, dietary habits, or sedentary lifestyle which drive them to develop T2DM.

Individuals with T2DM likely carry the genetic alleles that make them insulin resistance at different sites like liver, muscle, or adipose tissue. The genetic and epigenetic of T2DM had been explored since 2007 when the 5 whole genome-wide association studies were published on the genetics of T2DM.^3^ Unfortunately, there are few interventions could be offered once the discovery of genetic or epigenetic mutation which make the genetic testing less popular. Furthermore, T2DM is mostly polygenic disease. Apart from the documented monogenic causes, the contribution of different variant mutations in the inheritance of T2DM does not have great impact alone.^1^

Overall, T2DM is a complex syndrome. The role of genetic prediction of T2DM is still developing. Further studies on diabetes genetic mapping and their impact on clinical outcome are needed.

***Reply from the Author***

The first two third of the correspondence mentioned facts on the etiology of diabetes that are already described in details in the introduction and discussion parts of the article published by Yousuf et al.^1^

In human genetic association studies, cases group is selected based on the presence of the studied phenotype (in this case diabetes) and the control group is selected based on the absence of the studied phenotype (absence of diabetes=normal blood sugar based on fasting blood glucose and level of HbA1c). People in such studies try their best to match the 2 groups in gender and age while maintaining randomness and voluntary participation. In Yousuf et al^1^ number of male subjects in diabetic group were 63 and female 55 diabetic group. Whereas in the control group, male subjects were 58 and female subjects were 48. The Chi-square statistic is 0.0396. The *p*-value is 0.842272. The result is not significant at *p*<0.05. With respect to age, the mean age (±SD) was 53.19 (10.96) for the cases and 51.34 (13.78) for the control group. The t-test statistics= t=1.1172, df=222. The *p*-value is 0.2651. The result is not significant at *p*<0.05. In the published article, authors mistakenly reported a *p*-value of 0.002.

As mentioned in the correspondence and by Yousuf et al,^1^ diabetes is multifactorial in its etiology that include both genetic factors and environmental factors. Among the environmental factors are sugar intake, tobacco use, level of physical activity, stress, sleep deprivation, obesity and many others. In human genetic association studies, it is impossible to control for all such factors. Therefore, the BMI point raised in the correspondence is also not valid. With respect to sample size limitation, authors in Yousuf et al^4^ have discussed that in detail in the discussion section.

It is worth to mention that the common thing in genetic association studies is inconsistency in the findings due to genetic background, environmental factors, and criteria used in the diagnosis of complex diseases. Some alleles showed even opposite effects in different ethnicities. All such factors were discussed in Yousuf et al^4^ article.

The correspondence did not add much to that presented in the introduction and discussion section of the published article. The information regarding age and gender are not correct and should be corrected.

## References

[1] Yousuf AM , Kannu FA , Youssouf TM , Alsuhaimi FN , Aljohani AM , Alsehli FH , et al. Lack of association between fat mass and obesity-associated genetic variant (rs8050136) and type 2 diabetes mellitus. Saudi Med J 2022; 43: 132–138. 35110337 10.15537/smj.2022.43.2.20210822 PMC9127913

[2] Defronzo RA. Banting Lecture. From the triumvirate to the ominous octet: a new paradigm for the treatment of type 2 diabetes mellitus. Diabetes 2009; 58: 773–795. 19336687 10.2337/db09-9028 PMC2661582

[3] Groop L , Lyssenko V. Genes and type 2 diabetes mellitus. Curr Diab Rep 2008; 8: 192–197. 18625115 10.1007/s11892-008-0033-y

[4] Goodrich JK , Singer-Berk M , Son R , Sveden A , Sveden A , Wood J , et al. Determinants of penetrance and variable expressivity in monogenic metabolic conditions across 77,184 exomes. Nat Commun 2021; 12: 3505. 34108472 10.1038/s41467-021-23556-4 PMC8190084

